# Associations between school- and household-level water, sanitation and hygiene conditions and soil-transmitted helminth infection among Kenyan school children

**DOI:** 10.1186/s13071-015-1024-x

**Published:** 2015-08-07

**Authors:** M. C. Freeman, A. N. Chard, B. Nikolay, J. V. Garn, C. Okoyo, J. Kihara, S. M. Njenga, R. L. Pullan, S. J. Brooker, C. S. Mwandawiro

**Affiliations:** Department of Environmental Health, Emory University, Atlanta, Georgia USA; Faculty of Infectious and Tropical Diseases, London School of Hygiene and Tropical Medicine, London, UK; Eastern and Southern Africa Centre of International Parasite Control, Kenya Medical Research Institute, Nairobi, Kenya

## Abstract

**Background:**

Soil-transmitted helminths, a class of parasitic intestinal worms, are pervasive in many low-income settings. Infection among children can lead to poor nutritional outcomes, anaemia, and reduced cognition. Mass treatment, typically administered through schools, with yearly or biannual drugs is inexpensive and can reduce worm burden, but reinfection can occur rapidly. Access to and use of sanitation facilities and proper hygiene can reduce infection, but rigorous data are scarce. Among school-age children, infection can occur at home or at school, but little is known about the relative importance of WASH in transmission in these two settings.

**Methods:**

We explored the relationships between school and household water, sanitation, and hygiene conditions and behaviours during the baseline of a large-scale mass drug administration programme in Kenya. We assessed several WASH measures to quantify the exposure of school children, and developed theory and empirically-based parsimonious models.

**Results:**

Results suggest mixed impacts of household and school WASH on prevalence and intensity of infection. WASH risk factors differed across individual worm species, which is expected given the different mechanisms of infection.

**Conclusions:**

No trend of the relative importance of school versus household-level WASH emerged, though some factors, like water supply were more strongly related to lower infection, which suggests it is important in supporting other school practices, such as hand-washing and keeping school toilets clean.

**Electronic supplementary material:**

The online version of this article (doi:10.1186/s13071-015-1024-x) contains supplementary material, which is available to authorized users.

## Background

Over one billion people are infected with soil-transmitted helminths (STH), with the greatest burden of disease falling on children and the poor [1–3]. Safe and inexpensive drugs are available to treat STHs [4], and national deworming programmes are being rolled out in many parts of sub-Saharan Africa and Asia. However, in areas where mass drug administration (MDA) has been instituted, infection often occurs rapidly after treatment [5]. Environmental control of STH – specifically improving access to safe water supply, basic sanitation and improved hygiene (WASH) – may serve to complement these deworming efforts [6] and prove to be cost-effective. Recently there have been substantial commitments to bolster WASH control strategies for other so-called neglected tropical diseases such as trachoma [7], including large-scale implementation grants [8], but similar commitments have not been made for STH. This is in spite of the acknowledgement of WASH as a key control strategy for control of STH [9], and there has been little attention to developing relevant WASH targets [10]. Aside from the obvious cost implications of including WASH in STH control, few rigorous data exist to guide policy decisions on which interventions impact on STH infections and what indicators should be used to monitor progress.

Recent systematic reviews have found evidence of associations between STH infection and latrine access, shoe wearing, and hand-washing [11, 12]. Most of the data included in this review came from cross-sectional studies and the few quality randomized trials evaluating the impact of WASH on STH [12]. In Kenya, a school-based latrine construction and hygiene cluster-randomized trial found a 44 % reduction in the odds of *Ascaris lumbricoides* prevalence among those that received the WASH intervention and MDA, relative to MDA alone, though no effect was found for other worms [13]. Hand-washing behavior change trials in China and Peru have shown reductions in STH [14, 15], and a number of other large-scale trials that are currently underway may provide additional scientific, policy and programmatic evidence [16, 17].

While trials serve as the gold standard for assessing impact, they are costly and time consuming. Global STH mapping efforts have focused on publically available data to improve targeting for MDA [18, 19], however, WASH data from these studies are limited. Predictive water and sanitation maps on WASH coverage in sub-Saharan Africa have been recently developed [20]. While the use of these predicted coverage estimates for programme purposes will prove useful, quantitative estimates of the impact of WASH on STH remain elusive.

There are several additional limitations common across the WASH and STH literature. First, the majority of these studies do not use consistent measures of WASH. Second, in many of the published studies, WASH is not the main outcome of interest, and is only included in multivariable models when statistically significant, potentially biasing meta-analyses that use adjusted estimates of effect. Finally, most of the data estimating the association between WASH and STH capture only household conditions, ignoring other potential infectious environments, such as schools. The result is that the ability to harmonize lessons in terms of impact and cost-effectiveness of WASH improvements on STH from these studies is limited [12].

We conducted a cross-sectional study, embedded in the monitoring and evaluation of the Kenyan National School-Based Deworming Program [21], to assess the role of school and household-based WASH infrastructure and behaviours on STH infection prior to any mass drug administration. We assessed various approaches to measuring WASH exposures faced by school children to develop a theory- and empirically-based parsimonious set of variables for modeling. We report on baseline measures of association, prior to deworming, taking advantage of the natural heterogeneity in school WASH throughout Kenya, and the variability of household access and behaviours among children at the same school. Understanding the impact of WASH conditions both at home and school, where children spend a considerable part of their day, may provide insight into the public versus private domains of transmission [22], as well as addressing whether improved conditions in one domain are necessary or sufficient to reduce infection. The purpose of this study was to provide information about important indicators that could be used to monitor WASH and to understand the relative importance of school and household WASH conditions on the risk of STH species infection.

## Methods

### Ethics statement

Data collection for this study was approved by the Kenya Medical Research Institute (KEMRI) Ethics Review Committee (SSC 2206). Additional approvals were provided by the appropriate County-level health and education authorities. Prior to conducting study activities in each school, head teachers were asked to inform the students, parents and the school committee members about the survey and obtain their approval for the study. Parents/guardians who did not want their children to participate in the study were free to refuse participation. On the survey day, the survey team informed all children in the school about the sampling and survey procedures, making it clear that their participation was voluntary and that they may opt out of the testing at any time. This opt-out approach to consent was considered to be an ethical and practical way of informing participants in low-risk studies and interventions.

### Study design

We conducted an exploratory, cross-sectional analysis to assess the relative contribution of individual WASH characteristics at home and school-level on STH infection among Kenyan school children, prior to any MDA. Specifically, we examined data from Kenya’s National School-Based Deworming Program, which utilizes repeated cross-sectional surveys to assess the impact of school-based deworming on helminth infection among pupils [21]. In the deworming programme, data on STH infection were collected from over 20,000 children enrolled in 200 schools in STH endemic areas between January and April 2012. From the sample of 200 schools, we collected additional data on school water, sanitation and hygiene (WASH) access in 70 schools that had previously been selected for pre- and post-treatment parasitological examination (60 schools) or as a “high-frequency” sample in which students were followed longitudinally over the study period (10 schools). Previous large-scale MDA in Kenya had not been conducted since 2009 [21]. Though localized drug distribution may have taken place since, the baseline prevalence of infection in this study indicates they were not regular or widespread. For additional detail on the sampling approach, see Mwandawiro et al. [21].

### Survey procedures

In each school, nine boys and nine girls from the Early Childhood Development (pre-school) class and classes two through six were randomly selected to provide stool samples prior to deworming (totaling 108 pupils per school). Two separate slides were prepared and examined for the presence and intensity of STH species (hookworm *spp.*, *A. lumbricoides*, *T. trichiura*) using the Kato Katz method. Presence of infection for each worm species was defined as detection of one or more eggs on either slide. Infection intensity was estimated by averaging eggs per gram (epg) of faeces on both slides.

From the sample of students selected to provide stool specimens, students in classes two through six were randomly selected for a pupil interview. Equal numbers of boys and girls were selected; we aimed to select 50 students from high-frequency schools and 90 students from pre- and post-treatment parasitological examination schools, however, the actual sample size in each school was slightly less due to logistical reasons, such as there not being enough students present from a given class and sex. Using a structured paper-based questionnaire, trained enumerators interviewed selected pupils about their school and household WASH access and practices. Data were collected on age, sex, number of household residents and household assets. Climate, ecology, and population density covariates were compiled from a variety of sources and linked to school locations [21].

### WASH exposures

WASH covariates of interest included individual, household, and school-level variables. Individual-level variables included observed shoe-wearing and reported soil-eating practices (also known as pica or geophagy), a common practice in Kenya that has been previously associated to STH infection [23]. Students reported on their defecation and urination behaviours at school and at home. Household-level variables derived from pupil interviews included the type of water source; availability of a toilet/latrine; presence of a hand washing facility; and availability of water for hand-washing, soap for hand-washing, and tissue or water for use after defecation. Student-reported household availability of water for hand-washing, soap for hand-washing, and tissue or water for use after defecation were dichotomized as never/sometimes versus always available. Structured observations at each school were used to complement student-reported school WASH access and to record the sanitation conditions of school latrines, and were aggregated at the school level. School-level observations and pupil-reported conditions and behaviours included type of water source; availability of drinking water; type of latrines; pupil per latrine ratio; availability of hand-washing facilities, water for hand-washing, soap for hand-washing; availability of tissue or water for use after defecation; latrine condition. All student-reported school WASH access variables (availability of drinking water, water for hand-washing, soap, and tissue or water for after defecation) were aggregated at the school level.

We used several approaches to reduce our WASH predictors prior to model specification. When we had variables measuring a similar construct, we assessed pairwise relationships using Pearson correlations (*p* < 0.05). We assessed various pupil self-reported measures for WASH behaviours at home, at school, and for school conditions. In addition, we assessed if pupil reported conditions at school were reliable predictors of observed conditions. Where possible, we used parameters that we perceived to be more objective measures of conditions at school and in the household. This process was both useful for our modeling approach, but also for continued refinement of parsimonious follow-up surveys.

We created variables between 0 and 1 representing the availability of household and school hand-washing facilities with soap and water. A score of 0 indicated that the household/school did not have a pupil-reported hand-washing facility, and water and soap for hand-washing were reported as never or sometimes available. A score of 1 indicated that the household/school had a pupil-reported hand-washing facility and water and soap were reported as always available. The aggregated values of school tissue/water availability and drinking water availability were dichotomized as never/sometimes versus always available, and included in the models in place of the aggregated continuous variables, which would not converge in the negative binomial models. A pupil per latrine variable was created by dividing the number of pupils in the school by the number of functional latrines recorded during structured observation. School toilets were classified as being ventilated improved pit (VIP) latrines (10.4 %) – those that have a pipe and fly screen – compared to any other sanitation facility, most of which were traditional pit latrines (87.1 %).

We utilized factor analysis [24] to create a set of variables to measure school latrine sanitation conditions and to control for socio-economic status. Factors with Eigenvalues greater than one were retained, and factor values were calculated. Variables used for index calculation included in the school latrine sanitation factor analysis included observations on the functionality of latrine doors; structural condition of the slabs, walls, and roofs; presence of visible faeces, flies, and excessive smell; and whether the latrine tank was full. Of these, two factors were retained, determined predominately by the cleanliness and the structural integrity of the school latrines, where a higher factor score indicated better latrine cleanliness/structure.

Household assets used to estimate socio-economic status included wall, floor, and roof construction materials in the pupil’s house, and ownership of electricity and a mobile phone. Of these, eight factors were retained as control variables in the models.

We employed factor analysis to identify key climate variables to control for in our final models. Twelve variables measuring climate, ecology, and population density were included in the climate factor analysis: minimum, maximum, and mean monthly temperature; annual precipitation; altitude; monthly minimum, maximum, and mean vegetation coverage, measured by the Normalized Difference Vegetation Index (NDVI); Euclidian distances to a river and to any water body; population density; and land cover. Four variables explaining the most variability in the data were retained: mean temperature, annual precipitation, mean NDVI, and population density.

### Statistical analysis

In order to maximize the sample included in analysis, 168 missing values were imputed. Observations missing at the individual level, such as household latrine (*n* = 65), were imputed as the mean latrine availability based on other students’ responses in respective participants’ schools. Missing school-level WASH characteristics (e.g. presence of hand-washing (*n* = 9), water (*n* = 7) and soap (*n* = 7)) were derived from school-level mean estimates. One school was missing a response for the observed presence of excessive smell in functional school latrines; this value was imputed based on the same observation made during the second round of data collection so as not to lose data from an entire school. Models with imputed observations were compared to models excluding missing observations and no significant differences in model results were observed, thus we present the imputed results.

We conducted exploratory models of the associations between school and household WASH access and practices on infection with STH. Our two outcomes of interest were presence and intensity of STH species. Presence of infection was examined for each individual species and for any STH infection (defined as presence of at least 1 STH species). To assess presence we used univariable (unadjusted) and multivariable (fully adjusted) mixed effects logistic regression, with a random intercept to account for clustering of pupils within the school. We conducted univariable analyses of STH prevalence, stratified by province. While we do present the associations between WASH and any STH infection, this is largely due to the policy relevance of this association; the relationship between individual species and WASH may be more relevant biologically, given the different life cycles and exposure pathways for different species. We present the results stratified by county, given that each region represents different climatic, cultural, and environmental conditions that influence transmission patterns and pathways.

A common proxy of infection intensity is the number of eggs per gram of faeces for each species. Due to over-dispersed data, to assess infection intensity we used mixed effects negative binomial regression, with a random intercept to account for clustering of pupils within the school. We did not stratify the infection intensity analyses due to an inability of many of the models to converge.

All multivariable models controlled for region, pupil demographics (age, sex, number of people living in the pupil’s household, socio-economic status); school climate; and the number of students in the pupil’s school. Data were cleaned and analysed using STATA version 13 (STATA Corporation, College Station, TX, USA). Prevalence of *T. trichiura* infection was low and focal, and the measures of association were therefore imprecisely measured; as such, we don’t report the associations in the main body of the text (See Additional file 1: Table S1).

## Results

### Determining covariates of interest

We found that asking children to self-report if they had a toilet at home was highly correlated with where they reportedly “usually” go to defecate (p < 0.001) and urinate (p < 0.001). Statistical associations were found between reported household toilet, place to wash hands after defecation, water availability at that place, availability of soap for hand-washing at that place, and tissue/water availability for anal cleansing (all pairwise correlations p < 0.001). We retained three different measures of household sanitation and hygiene: reported presence of the toilet facility; availability of a hand-washing facility with soap and water, as it is a more comprehensive measure of hygiene, and availability of tissue/water for anal cleansing, since we were interested in this determinant in the predominantly Muslim population in the Coast region.

Children also provided similar answers to where they “usually” defecate, “usually” urinate, “last” defecated, and “last” urinated at school (all pairwise correlations p < 0.001). Reported home and school urination, as well as home and school defecation patterns were similarly answered (all pairwise correlations, p < 0.001), meaning that pupils who reported defecation/urination at school similarly reported those behaviours at home. Observed pupil to latrine ratio was associated with both reported school “usual” defecation behaviours (p < 0.04), but not urination patterns (p = 0.87). We perceived pupil reported presence of a toilet at home and school-based observations as more objective measures than pupil-reported defecation patterns; as such, we retained the presence of home and school latrines as covariates in our multivariable models. Given the limited applicability of urination patterns in the transmission of STH, we did not include self-reported urination patterns in the model.

Pupil reported presence of school hand-washing stations, soap availability, hand-washing water availability were all well correlated (all pairwise correlations p < 0.001). However, school-level observations of drinking water and hand-washing water were not statistically correlated (p > 0.15). The combined index of school-based measures was less reliable than those in the household (α = 0.25). We assessed the correlation between proportions of pupil reported conditions at school and observed conditions on the day of the visit. The observed presence of soap was well correlated with percentage of students who reported soap was “always” present (p < 0.001), although 470 (9.6 %) of students reported that soap was “never” available for hand-washing when it was observed the day of the visit and 266 (5.4 %) reported it was “always” available when it was not observed. The proportion of students that reported that drinking water was always available was also correlated with observed water at the school (p < 0.05). Since pupil-reported measures (average of all students measured) of soap, drinking water, hand-washing water, and tissue present for anal cleansing provide a quantitative measure that may more accurately reflect observations on a single day of visit, we retained those in the model. While this measure requires pupil interviews, it is likely more robust to the typical conditions at the school.

### Study population and household and school WASH characteristics

**Fig. 1:**
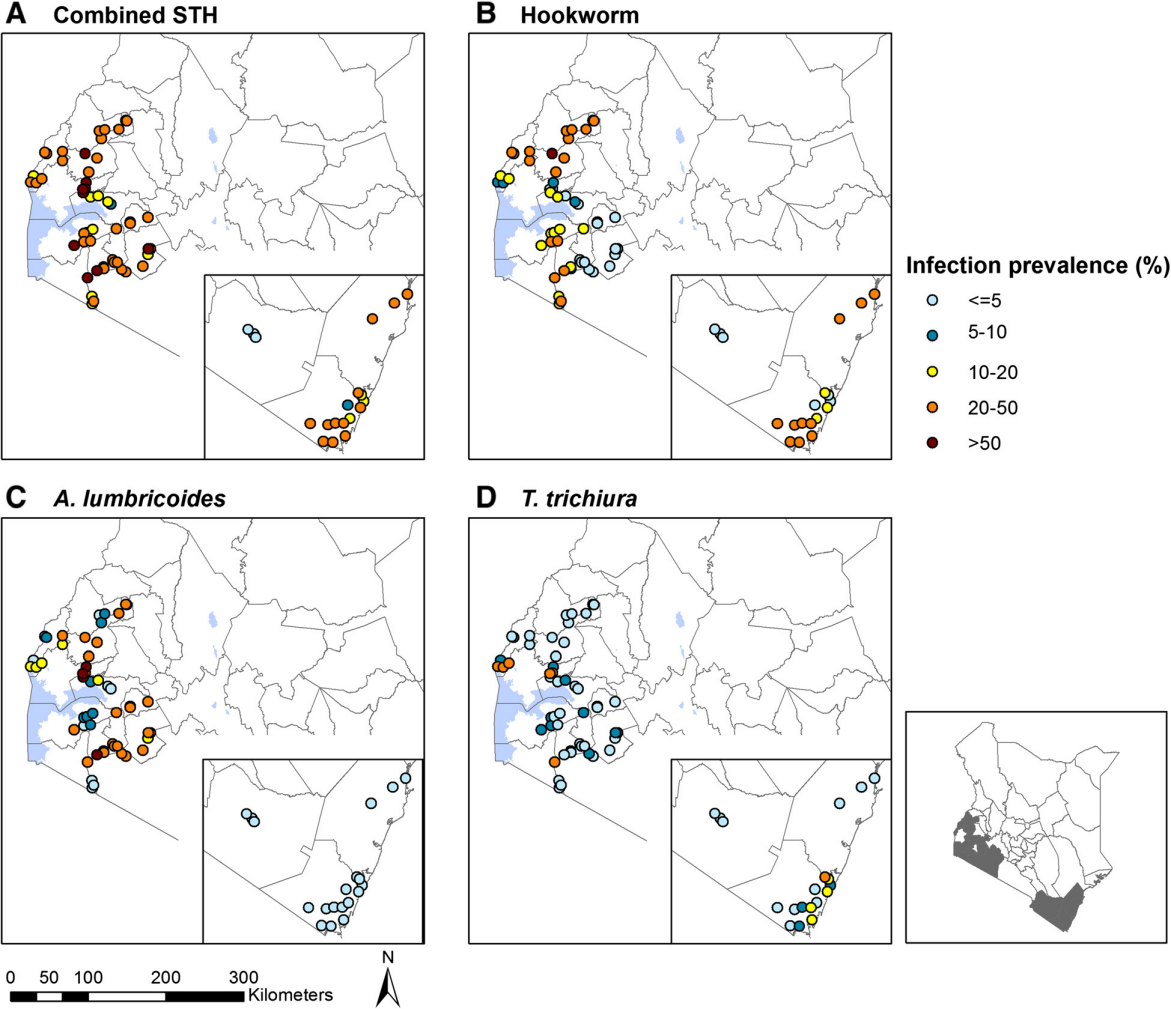
Prevalence of infection for combined helminth infection and individual species

We had WASH survey data and STH infection data from 4,931 students from 70 schools across four regions (Table 1; Fig. 1). Overall, 1,614 (32.7 %) children were infected with at least one STH species. The most prevalent single infection was *A. lumbricoides* (17.9 %). Geometric means of infection intensity were 153.3 epg for hookworm, 2754.5 epg for *A. lumbricoides*, and 94.4 epg for *T. trichiura*. Western region had the highest prevalence of any STH infection, as well as hookworm infection, and *T. trichiura* infections and infection intensities. Rift Valley had the highest prevalence of *A. lumbricoides* infection and the highest *A. lumbricoides* infection intensity.

**Table 1:** Pupil, household, and school WASH characteristics overall and stratified by county, Kenya, January-April 2012

	Overall (*n* = 4,931)	Coast (*n* = 1,305)	Rift Valley (*n* = 723)	Western (*n* = 1,526)	Nyanza (*n* = 1,377)
	N (%) or mean (SD)	N (%) or mean (SD)	N (%) or mean (SD)	N (%) or mean (SD)	N (%) or mean (SD)
STH infection					
Any STH infection	1,614 (32.7 %)	304 (23.3 %)	263 (36.4 %)	631 (41.4 %)	416 (30.2 %)
Hookworm prevalence	824 (16.7 %)	240 (18.4 %)	16 (2.2 %)	374 (24.5 %)	194 (14.1 %)
Hookworm infection intensity^¶^	153.3	93.5	111.1	360.6	55.8
*A. lumbricoides* prevalence	880 (17.9 %)	14 (1.1 %)	232 (32.1 %)	381 (25.0 %)	253 (18.4 %)
*A. lumbricoides* infection intensity^¶^	2754.5	144.1	4012.8	2972.6	2047.8
*T. trichiura* prevalence	302 (6.1 %)	90 (6.9 %)	44 (6.1 %)	132 (8.7 %)	36 (2.6 %)
*T. trichiura* infection intensity^¶^	94.4	45.2	127.6	184.7	35.1
Individual and household characteristics					
Girl	2,508 (50.9 %)	683 (52.3 %)	363 (50.9 %)	771 (50.5 %)	691 (50.2 %)
Age	10.8 (2.1)	10.9 (2.1)	10.8 (2.2)	10.8 (2.1)	10.7 (2.0)
Number of people living pupil’s household	6.9 (2.3)	6.8 (2.4)	7.2 (2.2)	6.6 (2.2)	7.2 (2.2)
Shoe-wearing	2,307 (46.9 %)	687 (52.8 %)	387 (53.7 %)	571 (37.5 %)	662 (48.2 %)
Soil-eating behaviour	838 (17.2 %)	131 (10.1 %)	71 (9.8 %)	237 (15.5 %)	399 (29.0 %)
Improved water source	2,692 (54.7 %)	998 (76.7 %)	187 (25.9 %)	788 (51.7 %)	719 (52.3 %)
Toilet/latrine available	4,123 (84.7 %)	974 (75.0 %)	647 (89.7 %)	1,425 (93.7 %)	1,077 (81.3 %)
Hand-washing facility with soap and water always available	1,373 (27.9 %)	359 (27.6 %)	209 (29.2 %)	365 (24.0 %)	440 (32.0 %)
Tissue/water for anal cleansing always available	2,259 (45.9 %)	991 (76.1 %)	340 (47.1 %)	363 (23.8 %)	565 (41.1 %)
School characteristics	Overall (n = 70)	Coast (n = 19)	Rift Valley (n = 10)	Western (n = 21)	Nyanza (n = 20)
Number of children in school	605.3 (339.5)	562.7 (456.2)	474.9 (209.6)	819.6 (262.3)	485.8 (227.3)
Improved water source	54 (77.1 %)	17 (89.5 %)	9 (90.0 %)	16 (76.2 %)	12 (60.0 %)
VIP latrine	7 (10.0 %)	4 (21.1 %)	0 (0.0 %)	0 (0.0 %)	3 (15.0 %)
Pupils per latrine	47.1 (39.6)	41.8 (29.1)	54.0 (48.3)	44.0 (22.8)	51.9 (56.0)
Hand-washing facility with soap and water always available^§^	2 (2.9 %)	0 (0.0 %)	0 (0.0 %)	0 (0.0 %)	2 (10.0 %)
Drinking water always available^§^	15 (21.4 %)	2 (10.5 %)	1 (10.0 %)	8 (38.1 %)	4 (20.0 %)
Tissue/water for anal cleansing always available^§^	4 (5.7 %)	4 (21.1 %)	0 (0.0 %)	0 (0.0 %)	0 (0.0 %)
Latrine sanitation: cleanliness^*^	0.001 (0.97)	0.15 (0.59)	−0.82 (2.08)	−0.03 (0.61)	0.31 (0.36)
Latrine sanitation: structural integrity^*^	−0.04 (1.00)	0.05 (0.93)	−0.59 (1.00)	0.59 (0.95)	−0.51 (0.73)

^¶^ measured using eggs per gram of faeces and displayed as geometric mean; ^§^school-aggregated proportion of pupil-reported characteristics “always” available; ^*^A higher score indicates better cleanliness/structural integrity;

The mean age of students was 10.9 years (SD 2.08 years), with ages ranging from 5 to 21 years. Over half of the students (53.1 %) were not wearing shoes at the time of the interview, and geophagy was reported among 17.2 % of students. Over half (54.7 %) of students’ reported use of an improved water source for drinking, and 84.4 % of students had a household sanitation facility. Fewer than half of students reported always having a hand-washing facility equipped with water and soap (27.9 %), or tissue/water for anal cleansing (45.9 %) available in their households.

**Fig. 2:**
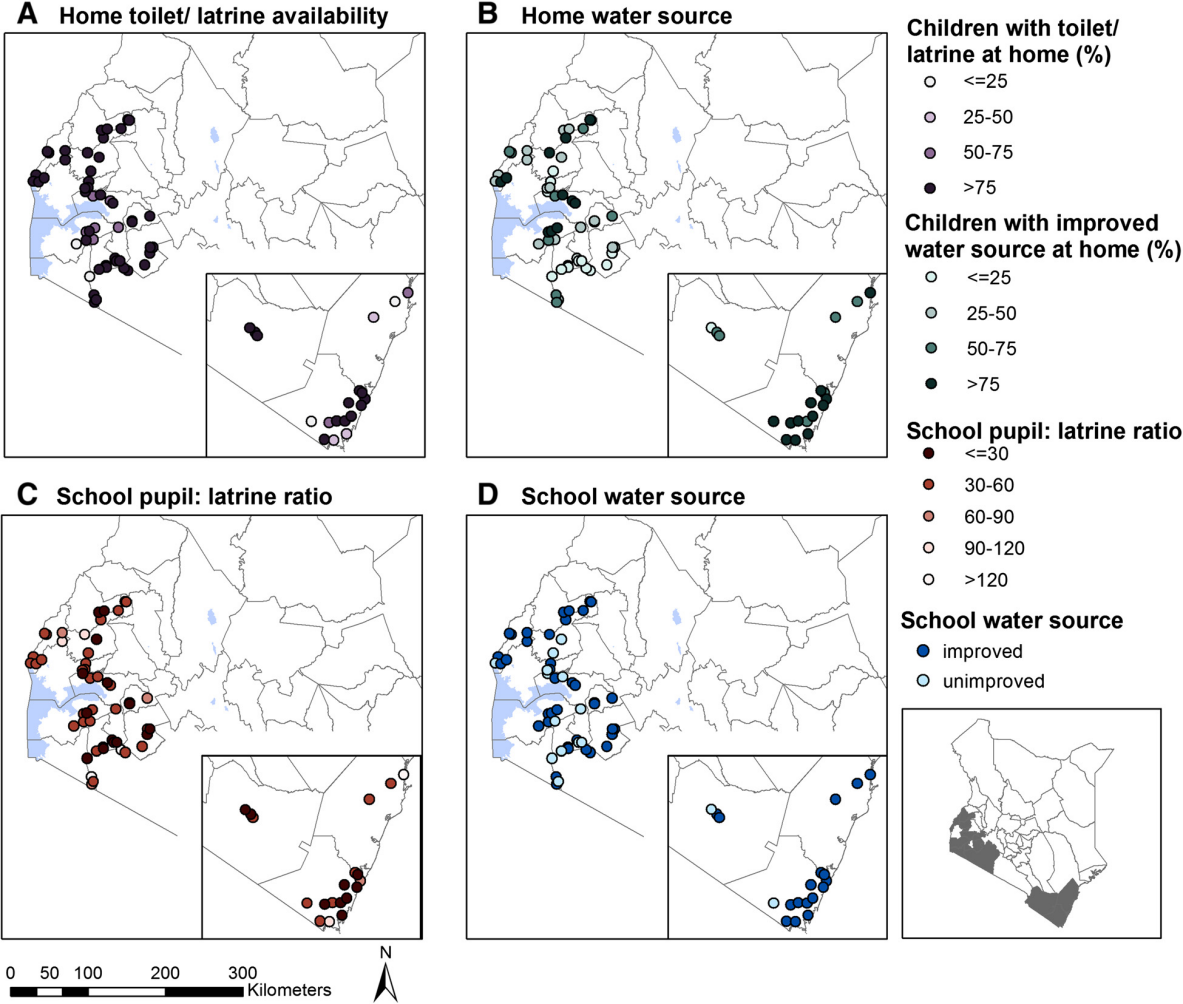
Prevalence of school and household water and sanitation conditions

School WASH conditions varied considerably by county/region (Table 1; Fig. 2). Improved water sources were observed in 77.1 % of schools. The mean pupil per latrine ratio was nearly 50:1, and 47 (67.1 %) schools exceeded the Government of Kenya’s (GoK) standard of 30 pupils per latrine [25]. Hand-washing facilities with soap and water were reported to be always available in just two schools (2.9 %). Drinking water and tissue/water for anal cleansing were 21.4 % and 5.7 % of schools, respectively.

### Univariable analysis

Univariable tests of association revealed significant associations between helminth prevalence/infection intensity and many of our covariates of interest, but few patterns existed across regions, by WASH classification (water, sanitation or hygiene), between school and home, or between worm species (Table 2). Associations between WASH covariates and worm prevalence and epg are found in Additional file 2: Table S2 and Additional file 3: Table S3. We found that stronger evidence that WASH covariates were associated with eggs per gram of feces than prevalence measures for each worm species.

**Table 2:**
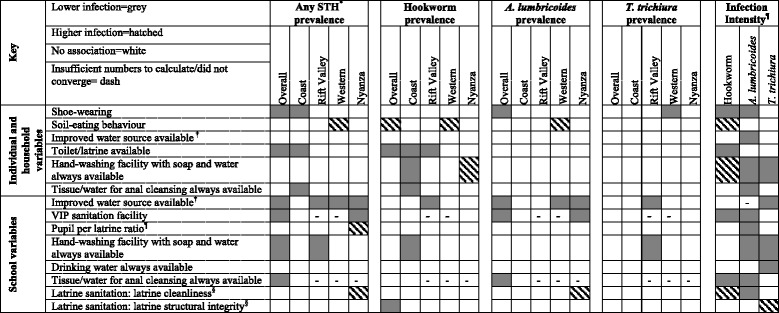
Univariable associations between WASH conditions and STH infection among school children in Kenya, 2012 (*n* = 4,931)

^*^Defined as infection by at least one of the following: hookworm, *A. lumbricoides*, *T. trichiura.* ^†^Improved sources are defined by the UNICEF/WHO joint monitoring programme (wssinfo.org). ^¶^OR and IRR represents the change in infection for each ten unit increase in a school’s pupil per latrine ratio. ^§^Higher score indicates greater cleanliness/structural integrity. Grey color or hatch fill indicates a significant association between groups at *p* < 0.05, as shown in the key above. *p*-values for prevalence estimates based on random effects logistic regression; p-values for infection intensity estimates based on random effects negative binomial regression. ^¶^ Measured using eggs per gram of faeces

### Multivariable analysis

Pupils who were observed wearing shoes at the time of the survey and who reported having a toilet or latrine at home had lower odds of having any STH infection (Table 3). Pupils with more frequent access to hand-washing facilities equipped with soap and water were more likely to be infected with STH. Attending a school with a VIP latrine, more frequent access to hand-washing facilities equipped with soap and water, and more frequent access to tissue/water for anal cleansing yielded lower odds of having any STH infection (Table 3).

**Table 3:** Multivariable associations between WASH conditions and STH infection^a^ among school children in Kenya, 2012 (*n* = 4,931)

	OR	95 % CI	p
Individual and household variables			
Shoe-wearing	***0.85***	***0.73, 0.99***	***0.03***
Soil-eating behaviour	1.11	0.91, 1.34	0.29
Improved water source available^†^	0.92	0.78, 1.08	0.31
Toilet/latrine available	***0.75***	***0.60, 0.93***	***<0.01***
Hand-washing facilities with soap and water availability	***1.22***	***1.01, 1.48***	***0.04***
Tissue/water always available for anal cleansing	0.97	0.83, 1.15	0.76
School variables			
Improved water source available^†^	0.66	0.43, 1.00	0.05
VIP sanitation facility	***0.53***	***0.31, 0.90***	***0.02***
Pupil per latrine ratio^¶^	0.98	0.94, 1.02	0.36
Hand-washing facility with soap and water availability	***0.57***	***0.35, 0.92***	***0.02***
Drinking water always available	0.95	0.66, 1.38	0.80
Tissue/water always available for anal cleansing	***0.36***	***0.16, 0.82***	***0.01***
Latrine sanitation: latrine cleanliness^§^	0.99	0.85, 1.15	0.87
Latrine sanitation: structural integrity^§^	0.88	0.74, 1.03	0.12

^a^Defined as infection by at least one of the following: hookworm, *A. lumbricoides*, *T. trichiura.* ^†^Improved sources are defined by the UNICEF/WHO joint monitoring programme (wssinfo.org). ^¶^OR and IRR represents the change in infection for each ten unit increase in a school’s pupil per latrine ratio. ^§^Higher score indicates greater cleanliness/structural integrity. ***Bold italicized*** associations indicate a significant association at *p* < .05. *p*-values based on random effects logistic regression (infection) and random effects negative binomial regression (infection intensity). Models control for province, pupil demographics (age, sex, number of people living in the pupil’s household, and household wealth); climate/ecology (temperature, precipitation, land cover, population density); and the number of students in the pupil’s school

Geophagy and frequency of available household hand-washing facilities with soap and water were associated with significantly greater odds of hookworm infection and significantly higher hookworm infection intensity (Table 4). Household toilet/latrine access was associated with lower odds of hookworm infection and a lower rate of infection intensity. Hookworm infection was not significantly associated with any school-level variables. However, students attending schools with VIP sanitation facilities and more frequent availability of tissue/water for anal cleansing had significantly lower rates of infection intensity, while students attending schools with higher pupil to latrine ratios and latrines exhibiting greater cleanliness had significantly higher rates of infection intensity.

**Table 4:** Multivariable associations between WASH conditions and hookworm infection and infection intensity among school children in Kenya, 2012 (*n* = 4,931)

	Infection			Infection Intensity		
	OR	95 % CI	p	IRR	95 % CI	p
Individual and household variables						
Shoe-wearing	0.96	0.79, 1.17	0.69	0.96	0.82, 1.11	0.58
Soil-eating behaviour	***1.31***	***1.03, 1.66***	***0.03***	***1.36***	***1.13, 1.63***	***<0.01***
Improved water source available^†^	1.01	0.82, 1.23	0.95	1.03	0.87, 1.22	0.74
Toilet/latrine available	***0.67***	***0.51, 0.87***	***<0.01***	***0.64***	***0.52, 0.78***	***<0.01***
Hand-washing facilities with soap and water availability	***1.41***	***1.09, 1.83***	***<0.01***	***1.37***	***1.11, 1.69***	***<0.01***
Tissue/water always available for anal cleansing	0.90	0.73, 1.11	0.32	0.94	0.79, 1.12	0.48
School variables						
Improved water source available^†^	1.08	0.52, 2.24	0.83	0.90	0.70, 1.16	0.42
VIP sanitation facility	0.54	0.22, 1.32	0.18	***0.61***	***0.44, 0.86***	***<0.01***
Pupil per latrine ratio^¶^	1.01	0.94, 1.08	0.84	***1.02***	***1.00, 1.05***	***0.05***
Hand-washing facilities with soap and water availability	0.48	0.21, 1.10	0.08	0.81	0.60, 1.10	0.18
Drinking water always available	0.95	0.50, 1.79	0.86	0.93	0.76, 1.15	0.51
Tissue/water always available for anal cleansing	0.37	0.09, 1.48	0.16	***0.49***	***0.29, 0.81***	***<0.01***
Latrine sanitation: latrine cleanliness^§^	1.36	0.90, 2.05	0.15	***1.40***	***1.19, 1.64***	***<0.01***
Latrine sanitation: structural integrity^§^	1.02	0.76, 1.37	0.91	0.95	0.86, 1.05	0.31

^†^Improved sources are defined by the UNICEF/WHO joint monitoring programme (wssinfo.org). ^¶^OR and IRR represents the change in infection for each ten unit increase in a school’s pupil per latrine ratio. ^§^Higher score indicates greater cleanliness/structural integrity. *Bold italicized* associations indicate a significant association at *p* < .05. *p*-values based on random effects logistic regression (infection) and random effects negative binomial regression (infection intensity). Models control for province, pupil demographics (age, sex, number of people living in the pupil’s household, and household wealth); climate/ecology (temperature, precipitation, land cover, population density); and the number of students in the pupil’s school

Observed shoe-wearing, improved school water source, and better school latrine structural integrity was associated with significantly lower odds of *A. lumbricoides* infection and lower rates of infection intensity (Table 5). Household toilet/latrine availability and school VIP sanitation facilities were associated with significantly lower *A. lumbricoides* infection intensity. Infection intensity significantly decreased as latrine cleanliness increased and as pupil per latrine ratio increased.

**Table 5:** Multivariable associations between WASH conditions and *A. lumbricoides* infection and infection intensity among school children in Kenya, 2012 (*n* = 4,931)

	Infection			Infection Intensity		
	OR	95 % CI	p	IRR	95 % CI	p
Individual and household variables						
Shoe-wearing	***0.79***	***0.65, 0.97***	***0.03***	***0.77***	***0.66, 0.90***	***<0.01***
Soil-eating behaviour	1.12	0.87, 1.45	0.38	1.04	0.87, 1.25	0.67
Improved water source available^†^	0.94	0.77, 1.16	0.58	0.86	0.74, 1.01	0.07
Toilet/latrine available	0.83	0.60, 1.14	0.25	***0.71***	***0.56, 0.89***	***<0.01***
Hand-washing facilities with soap and water availability	1.05	0.82, 1.35	0.68	1.01	0.83, 1.23	0.89
Tissue/water always available for anal cleansing	0.97	0.77, 1.21	0.77	0.96	0.80, 1.15	0.66
School variables						
Improved water source available^†^	***0.45***	***0.22, 0.92***	***0.03***	***0.51***	***0.4, 0.64***	***<0.01***
VIP sanitation facility	0.38	0.12, 1.21	0.10	***0.36***	***0.17, 0.75***	***<0.01***
Pupil per latrine ratio^¶^	0.96	0.89, 1.03	0.22	***0.97***	***0.94, 0.99***	***0.01***
Hand-washing facilities with soap and water availability	0.69	0.31, 1.55	0.37	0.83	0.61, 1.13	0.23
Drinking water always available	0.97	0.51, 1.81	0.91	1.14	0.94, 1.39	0.19
Tissue/water always available for anal cleansing	0.19	0.02, 1.72	0.14	0.30	0.06, 1.43	0.13
Latrine sanitation: latrine cleanliness^§^	0.92	0.72, 1.19	0.54	***0.90***	***0.84, 0.97***	***<0.01***
Latrine sanitation: structural integrity^§^	***0.73***	***0.54, 0.99***	***0.04***	***0.81***	***0.73, 0.90***	***0.00***

^†^Improved sources are defined by the UNICEF/WHO joint monitoring programme (wssinfo.org). ^¶^OR and IRR represents the change in infection for each ten unit increase in a school’s pupil per latrine ratio. ^§^Higher score indicates greater cleanliness/structural integrity. *Bold italicized* associations indicate a significant association at *p* < .05. *p*-values based on random effects logistic regression (infection) and random effects negative binomial regression (infection intensity). Models control for province, pupil demographics (age, sex, number of people living in the pupil’s household, and household wealth); climate/ecology (temperature, precipitation, land cover, population density); and the number of students in the pupil’s school

## Discussion

We examined associations between school and household WASH infrastructure and pupil behaviour and the prevalence and intensity of STH species infection. We assessed these associations across four domains: 1) worm species, 2) region, 3) WASH exposures, and 4) individual-level, household-level, and school-level exposures. Improved WASH access was generally, but not always, associated with lower intensity of STH infection, though the relationships were not always consistent between worm species. We found fewer associations between improved WASH and prevalence of STH, and though many of our indicators of improved WASH suggested a protective effect of that exposure, there were few statistically significant findings. Few covariates were significantly associated with multiple outcomes, and results did not reveal clear patterns of association between WASH and STH infection across domains. We present data on the overall association between any STH infection and WASH, as this is how much of the policy around STH control is written. However, this analysis may obfuscate the true associations between WASH and individual STH species, especially given different pathways of exposure between species.

We set out to measure several ways of quantifying WASH exposures experienced by primary school children throughout Kenya. Several other studies have assessed the association between WASH and STH among children [26, 27], but few if any were conducted at scale or assessed both household and school-level covariates. The spatial distribution of STH infections in different regions in Kenya is well documented [28], and is driven by climatic factors (e.g., rainfall), environmental conditions (e.g., soil type and elevation), and living conditions (e.g. WASH access and building construction) that modify behaviours and exposure pathways. Co-infection with malaria may be another reason for geographic heterogeneity [29]. As such, describing the overall association between WASH and STH is important, but associations must also be considered within the context of the variability in geographic, climatic, and living conditions across regions [30, 31].

Objectively quantifying the access to and use of WASH infrastructure is challenging, and considerable debate exists on the right way to parameterize these exposures. Our approach of reducing variable selection using correlation and validating what may be perceived as subjective measures (pupil-reported data) with more objective measures (observed data) resulted in a more empirical approach to model selection. We found that children’s reports of school conditions closely matched observed conditions taken on a single day of the visit. While we utilized the pupil-reported covariates measuring the presence of water for drinking and hand-washing, soap, anal cleansing material, spot check observations may prove sufficient if the visits are made without informing the schools ahead of time. Several of the household conditions were important predictors of infection, and a basic set of WASH questions for future STH mapping activities would help to refine our understanding of the relative importance of household and school conditions in STH infections. Future studies could rely on fewer survey questions that focus on observation of WASH conditions at the school level, even if they are for only one point in time. Further, a substantial amount of information on household conditions and behaviours can be distilled from a few questions to pupils that focus on objective measures (e.g., presence of a toilet).

The presence of tissue paper or water for anal cleansing emerged as the most important predictor of STH infection. This association could be related to the religious beliefs and practices of the largely Muslim population in the Coast province. Although anal cleansing is not only restricted to Muslim cultures, Islamic tradition states that water - and tissue when necessary - should be used for anal cleansing after defecation. Consistent availability of anal cleansing materials was most common in households and schools in the predominantly Muslim coastal region. In the absence of tissue or water, anal cleansing after defecation is often done with one’s hand, which can directly lead to fecal contamination, and thus exposure to helminth infection. Without proper anal cleansing material, children may wipe their hands on latrine walls, which could increase pathogen exposure to others [32]. However, this would not explain increased infectivity due to the need for ova to embryonate in soil. Indeed, in the multivariable models, few of these associations held true: only pupils attending schools with more frequent availability of tissue or water for use after defecation had 64 % lower odds of any STH and lower rates of hookworm infection intensity.

As expected, several sanitation outcomes were strongly predictive of infection with different STH species, though the directions of these relationships were not always as anticipated. The presence of a household toilet was associated with lower STH prevalence, hookworm prevalence and intensity, and *A. lumbricoides* prevalence. These findings were commensurate with results from a recent systematic review that found approximately 40 % reduction in the odds of STH and individual worm species [12], though associations with intensity of infection have rarely been reported in the literature. Here we relied on pupil reported presence of a toilet, which may have biased our results towards the null, since children without toilets are more likely to report having a toilet than children with toilets are to report not having one. The positive relationship between household toilet and *T. trichiura* is somewhat inexplicable, unless it represents increased exposures due to poorly maintained facilities.

For school sanitation factors, the type of toilet, toilet conditions, and pupil to latrine ratio were all associated with overall or worm-specific infections, though these results did not produce a clear signal of an effect either for individual worms or for specific indicators. Having a VIP toilet was associated with lower STH prevalence and hookworm and *A. lumbricoides* infection intensity than having a traditional toilet. While better cleanliness and maintenance of school toilets and higher pupil to latrine ratios were associated with lower *A. lumbricoides* infection, these relationships were inverse for hookworm and non-existent for *T. trichiura*. Previous research suggests that some students are selective with latrine usage, and are less likely to use latrines that are in poor sanitary condition [33]. Thus, while it may appear that dirty latrines are protective against hookworm infection, it may be that students are simply not using the dirty latrines and therefore are not exposed to the pathway of hookworm infection. Equally likely is that our measure of latrine cleanliness is purely of superficial cleanliness, and may not measure the microbiological cleanliness that would impact on helminth infection.

We found that more frequent access to household hand-washing facilities equipped with soap and water increased the odds of any STH and hookworm infection and the rate of hookworm infection intensity. These results were surprising, as they are contrary to evidence suggesting that hand-washing after defecation and the availability or use of soap reduces the odds of STH infection [12]. When examining the stratified bivariate associations, access to household hand-washing facilities was associated with higher odds of hookworm infection in Nyanza, but lower odds of infection in the Coast Province. The pathway through which more frequent access to hand-washing facilities may increase the odds of hookworm infection is unclear and has limited biological plausibility given the transmission patterns of hookworm. However, unless pupils are actively practicing hand-washing, the mere presence of hand-washing facilities, soap, and water will not interrupt STH transmission. More research is needed to identify appropriate and reliable proxies for hand-washing behaviours, specifically to look at how sanitation, water, and hygiene characteristics interact to prevent exposure.

Pupils’ household drinking water source was not associated with STH infection outcomes, while pupils attending schools with improved water sources had lower *A. lumbricoides* prevalence and infection intensities. Based on the exposure patterns of STH, an improved drinking water source was not expected to be associated with reduced STH infection, especially since the designation of “improved” is not indicative of water quantity available for personal hygiene nor of water quality; however, access to improved sources at school likely supports better access to hand-washing water, water for anal cleansing, and cleaning of toilets.

At the individual level, we found that children wearing shoes had lower odds of any STH infection and of *A. lumbricoides* infection and infection intensity. This finding was initially surprising; previous literature has shown an association between shoe wearing and hookworm infection [12, 34, 35], as hookworm, unlike *A. lumbricoides*, can be transmitted by skin contact with infected soil. While our univariable tests of association indicated that shoe wearing was associated with lower hookworm infection intensity, shoe wearing was not significant in either of the multivariate models of hookworm infection. However, while wearing shoes may have no biological impact on *A. lumbricoides* infection, whether a child is wearing shoes may serve as a proxy for socio-economic status, thus corroborating previous research indicating that STH infection, particularly *A. lumbricoides,* is associated with lower socio-economic status [36, 37].

There are several limitations to this analysis. First, our analysis was exploratory and our findings of the WASH factors that were associated with STH are only as good as the measurements we used to assess those factors. Measuring WASH is a challenge, as the conditions, for example, access to soap, vary considerably over the course of the day or throughout the year. Measuring WASH access is also prone to bias. For this reason, we hope that our analysis will contribute to the ongoing discussion of indictors and metrics that can be used to support STH monitoring [38]. Second, since WASH conditions are highly associated e.g. improved water quantity supports greater hand-washing, a more nuanced analysis approach that accounts for complex interactions, such as conditional inference trees or classification and regression trees [39], may be a valuable contribution to research on the WASH and STH nexus.

## Conclusions

The purpose of our study was to capture the associations between WASH and STH in a holistic way that addressed different WASH exposures at school and at home, and assessed these relationships between geographic areas. We utilized data from Kenya’s National School-Based Deworming Program to evaluate the role of school and household-based WASH infrastructure and behaviours on STH infection among pupils prior to MDA. Results suggest mixed impacts of household and school WASH on prevalence and intensity of infection. WASH risk factors differed across individual worm species, which is expected given the different mechanisms of infection. No clear trend of the relative importance of school versus household-level WASH emerged, though some factors, like water supply seem to suggest the important role of school water supply, perhaps in supporting other school practices, such as hand-washing and keeping school toilets clean. Future research should investigate WASH indicators that are necessary and sufficient for infection by individual worm species. Additional analysis using approaches that account for complicated correlation between WASH factors is warranted.

## Additional files

Additional file 1: Table S1. Multivariable associations between WASH conditions and T. trichiura infection and infection intensity among school children in Kenya, 2012 (n=4,931). (DOCX 108 kb) Additional file 2: Table S2. Prevalence of STH by species for each WASH covariate among school children in Kenya, 2012 (n=4,931). (DOCX 125 kb) Additional file 3: Table S3. Mean EPG of faeces by STH species for each WASH covariate among school children in Kenya, 2012 (n=4,931). (DOCX 115 kb)
