# Supplementary material for: Associations between school- and household-level water, sanitation and hygiene conditions and soil-transmitted helminth infection among Kenyan school children
Source: Parasit Vectors. 2015 Aug 7;8:412. doi: 10.1186/s13071-015-1024-x (PMC4528701; doi:10.1186/s13071-015-1024-x)
Supplement: Additional file 1: Table S1. — Multivariable associations between WASH conditions and T. trichiura infection and infection intensity among school children in Kenya, 2012 (n=4,931). (DOCX 108 kb) [file 13071_2015_1024_MOESM1_ESM.docx]

| Table S1: Multivariable associations between WASH conditions and *T. trichiura* infection and infection intensity among school children in Kenya, 2012 (n=4,931) | | | | | | | | |
| --- | --- | --- | --- | --- | --- | --- | --- | --- |
|  | **Infection** | | |  | **Infection Intensity** | | |  |
|  | **OR** | **95% CI** | **p** |  | **IRR** | **95% CI** | **p** |  |
| **Individual and household variables** | | | | | | | | |
| Shoe-wearing | 0.79 | 0.58, 1.08 | 0.14 |  | 0.86 | 0.66, 1.11 | 0.25 |  |
| Soil-eating behaviour | 1.13 | 0.77, 1.64 | 0.53 |  | 1.21 | 0.88, 1.65 | 0.24 |  |
| Improved water source available^†^ | 1.00 | 0.71, 1.4 | 0.99 |  | 0.83 | 0.62, 1.11 | 0.20 |  |
| Toilet/latrine available | 1.51 | 0.98, 2.33 | 0.06 |  | ***1.51*** | ***1.05, 2.18*** | ***0.03*** |  |
| Hand-washing facilities with soap and water availability | 0.87 | 0.58, 1.31 | 0.51 |  | 0.83 | 0.58, 1.19 | 0.32 |  |
| Tissue/water always available for anal cleansing | 1.09 | 0.75, 1.57 | 0.66 |  | 1.07 | 0.77, 1.49 | 0.68 |  |
| **School variables** | | | | | | | | |
| Improved water source available^†^ | 0.41 | 0.16, 1.03 | 0.06 |  | ***0.35*** | ***0.23, 0.54*** | ***<0.01*** |  |
| VIP sanitation facility | 0.89 | 0.26, 3.00 | 0.85 |  | 1.67 | 0.94, 2.95 | 0.08 |  |
| Pupil per latrine ratio^¶^ | 1.00 | 0.91, 1.09 | 0.94 |  | 0.97 | 0.91, 1.03 | 0.32 |  |
| Hand-washing facilities with soap and water availability | 0.65 | 0.22, 1.91 | 0.44 |  | 0.93 | 0.52, 1.66 | 0.80 |  |
| Drinking water always available | 0.77 | 0.33, 1.81 | 0.55 |  | 0.78 | 0.52, 1.16 | 0.21 |  |
| Tissue/water always available for anal cleansing | 1.26 | 0.25, 6.22 | 0.78 |  | 0.86 | 0.41, 1.77 | 0.68 |  |
| Latrine sanitation: latrine cleanliness^§^ | 0.95 | 0.66, 1.35 | 0.76 |  | 0.83 | 0.68, 1.01 | 0.06 |  |
| Latrine sanitation: structural integrity^§^ | 0.94 | 0.66, 1.33 | 0.71 |  | 0.93 | 0.79, 1.09 | 0.36 |  |
| ^†^Improved sources are defined by the UNICEF/WHO joint monitoring programme (wssinfo.org). ^¶^OR and IRR represents the change in infection for each ten unit increase in a school’s pupil per latrine ratio. ^§^Higher score indicates greater cleanliness/structural integrity. ***Bold italicized*** associations indicate a significant association at p<.05. *p*-values based on random effects logistic regression (infection) and random effects negative binomial regression (infection intensity). Models control for province, pupil demographics (age, sex, number of people living in the pupil’s household, and household wealth); climate/ecology (temperature, precipitation, land cover, population density); and the number of students in the pupil’s school | | | | | | | |  |
