# Supplementary material for: Associations between school- and household-level water, sanitation and hygiene conditions and soil-transmitted helminth infection among Kenyan school children
Source: Parasit Vectors. 2015 Aug 7;8:412. doi: 10.1186/s13071-015-1024-x (PMC4528701; doi:10.1186/s13071-015-1024-x)
Supplement: Additional file 2: Table S2. — Prevalence of STH by species for each WASH covariate among school children in Kenya, 2012 (n=4,931). (DOCX 125 kb) [file 13071_2015_1024_MOESM2_ESM.docx]

| **Table S2.** Prevalence of STH by species for each WASH covariate among school children in Kenya, 2012 | | | | |
| --- | --- | --- | --- | --- |
|  | **Any STH (n=1,614)**  N (%) | **Hookworm (n=824)**  N (%) | ***T. trichiura* (n=302)**  N (%) | ***A. lumbricoides* (n=880)**  N (%) |
| **Pupil characteristics** | | |  |  |
| Shoe-wearing | | | | |
| No | 945 (36.2%) | 500 (19.1%) | 161 (6.2%) | 534 (20.4%) |
| Yes | 666 (28.9%) | 323 (14%) | 141 (6.1%) | 344 (14.9%) |
| Soil-eating behaviour | | | | |
| No | 1308 (32%) | 646 (15.8%) | 247 (6%) | 718 (17.6%) |
| Yes | 304 (36.3%) | 178 (21.2%) | 55 (6.6%) | 160 (19.1%) |
| **Household WASH characteristics** | | |  |  |
| Water source^†^ | | | | |
| Unimproved | 839 (37.6%) | 323 (14.5%) | 131 (5.9%) | 581 (26.0%) |
| Improved | 772 (28.7%) | 499 (18.5%) | 170 (6.3%) | 298 (11.1%) |
| Toilet/latrine available | | | | |
| No | 276 (37.1%) | 179 (24.1%) | 52 (7.0%) | 102 (13.7%) |
| Yes | 1320 (32.0%) | 635 (15.4%) | 248 (6.0%) | 771 (18.7%) |
| Hand-washing facility with soap and water availability | | | | |
| Never/sometimes | 1125 (33.0%) | 578 (16.9%) | 226 (6.6%) | 625 (18.3%) |
| Always | 482 (32.0%) | 245 (16.3%) | 75 (5.0%) | 250 (16.6%) |
| Tissue/water for anal cleansing availability | | | | |
| Never/sometimes | 956 (35.9%) | 472 (17.7%) | 173 (6.5%) | 592 (22.2%) |
| Always | 653 (28.9%) | 349 (15.4%) | 128 (5.7%) | 285 (12.6%) |
| **School characteristics** |  |  |  |  |
| Water source^†^ | | | | |
| Unimproved | 454 (43%) | 186 (17.6%) | 101 (9.6%) | 322 (30.5%) |
| Improved | 1160 (29.9%) | 638 (16.5%) | 201 (5.2%) | 558 (14.4%) |
| Toilet/latrine type | | | | |
| Traditional/water | 1537 (34.8%) | 767 (17.4%) | 278 (6.3%) | 872 (19.7%) |
| VIP | 77 (15.1%) | 57 (11.2%) | 24 (4.7%) | 8 (1.6%) |
| Pupils per latrine (ppl) | | | | |
| <30 ppl | 1050 (33.1%) | 567 (17.9%) | 206 (6.5%) | 561 (17.7%) |
| ≥30 ppl | 564 (32.0%) | 257 (14.6%) | 96 (5.4%) | 319 (18.1%) |
| Hand-washing facility with soap and water availability**^§^** | | | | |
| Never/sometimes | 1540 (32.9%) | 789 (16.8%) | 291 (6.2%) | 836 (17.8%) |
| Always | 70 (29.5%) | 34 (14.3%) | 11 (4.6%) | 41 (17.3%) |
| Drinking water availability**^§^** | | | | |
| Never/sometimes | 1265 (33.3%) | 627 (16.5%) | 253 (6.7%) | 679 (17.9%) |
| Always | 349 (30.7%) | 197 (17.3%) | 49 (4.3%) | 201 (17.7%) |
| Tissue/water for anal cleansing availability**^§^** | | | | |
| Never/sometimes | 1560 (33.8%) | 788 (17.1%) | 276 (6.0%) | 878 (19.0%) |
| Always | 54 (17.1%) | 36 (11.4%) | 26 (8.3%) | 2 (0.6%) |
| Latrine sanitation: cleanliness^*^ | | | | |
| 1^st^ quartile | 489 (39.1%) | 191 (15.3%) | 108 (8.6%) | 292 (23.4%) |
| 2^nd^ quartile | 390 (31.9%) | 228 (18.7%) | 58 (4.8%) | 226 (18.5%) |
| 3^rd^ quartile | 362 (28.6%) | 154 (12.2%) | 46 (3.6%) | 201 (15.9%) |
| 4^th^ quartile | 373 (31.2%) | 251 (21.0%) | 90 (7.5%) | 161 (13.5%) |
| Latrine sanitation: structural integrity^*^ | | | | |
| 1^st^ quartile | 416 (31.9%) | 209 (16.0%) | 57 (4.4%) | 235 (18.0%) |
| 2^nd^ quartile | 398 (31.2%) | 151 (11.8%) | 76 (6.0%) | 224 (17.6%) |
| 3^rd^ quartile | 380 (33.8%) | 227 (20.2%) | 79 (7.0%) | 181 (16.1%) |
| 4^th^ quartile | 420 (34.3%) | 237 (19.4%) | 90 (7.4%) | 240 (19.6%) |
| ^†^Improved sources are defined by the UNICEF/WHO joint monitoring programme (wssinfo.org). ^§^school-aggregated proportion of pupil-reported availability. ^*^A higher quartile indicates better cleanliness/structural integrity. | | | | |
