# Supplementary material for: Associations between school- and household-level water, sanitation and hygiene conditions and soil-transmitted helminth infection among Kenyan school children
Source: Parasit Vectors. 2015 Aug 7;8:412. doi: 10.1186/s13071-015-1024-x (PMC4528701; doi:10.1186/s13071-015-1024-x)
Supplement: Additional file 3: Table S3. — Mean EPG of faeces by STH species for each WASH covariate among school children in Kenya, 2012 (n=4,931). (DOCX 115 kb) [file 13071_2015_1024_MOESM3_ESM.docx]

| **Table S3.** Mean EPG of faeces by STH species for each WASH covariate among school children in Kenya, 2012 (n=4,931) | | | |
| --- | --- | --- | --- |
|  | **Hookworm**  mean (SD) | ***T. trichiura***  mean (SD) | ***A. lumbricoides***  mean (SD) |
| **Pupil characteristics** | | | |
| Shoe-wearing | | | |
| No | 75.5 (381.1) | 11.7 (72.4) | 1638.6 (5295.7) |
| Yes | 44.6 (351.6) | 12.6 (119.8) | 1339.5 (4999.6) |
| Soil-eating behaviour | | | |
| No | 60.6 (389.7) | 12.0 (101.0) | 1570.5 (5330.0) |
| Yes | 63.2 (233.2) | 12.8 (78.1) | 1139.4 (4209.5) |
| **Household WASH characteristics** | | | |
| Water source^†^ | | | |
| Unimproved | 55.0 (372.9) | 15.5 (117.9) | 2363.6 (6379.8) |
| Improved | 66.0 (363.5) | 9.3 (76.3) | 771.7 (3688.5) |
| Toilet/latrine available | | | |
| No | 56.3 (233.9) | 18.9 (162.9) | 1179.7 (4658.1) |
| Yes | 62.7 (389.4) | 11.0 (80.9) | 1560.3 (5249.9) |
| Hand-washing facility with soap and water availability | | | |
| Never/sometimes | 63.7 (338.7) | 14.5 (112.8) | 1490.8 (5108.1) |
| Always | 55.4 (427.3) | 6.6 (46.6) | 1500.4 (5252.7) |
| Tissue/water for anal cleansing availability | | | |
| Never/sometimes | 73.9 (376.7) | 17.4 (125.8) | 1725.0 (5414.5) |
| Always | 45.6 (356.4) | 5.9 (44.5) | 1230.0 (4830.9) |
| **School characteristics** | | | |
| Water source^†^ | | | |
| Unimproved | 58.9 (210.1) | 26.0 (155.1) | 2425.9 (6181.0) |
| Improved | 61.6 (399.9) | 8.3 (73.8) | 1242.9 (4808.8) |
| Toilet/latrine type | | | |
| Traditional/water | 62.5 (314.9) | 13.2 (102.6) | 1660.1 (5408.2) |
| VIP | 47.9 (668.2) | 2.9 (17.7) | 81.5 (1151.3) |
| Pupils per latrine (ppl) | | | |
| <30 ppl | 70.5 (437.9) | 12.7 (78) | 1439.8 (5074.8) |
| ≥30 ppl | 43.8 (180.9) | 11.1 (124.9) | 1598.6 (5299.3) |
| Hand-washing facility with soap and water availability**^§^** | | | |
| Never/sometimes | 63.1 (376.2) | 12.6 (99.8) | 1514.6 (5190.9) |
| Always | 20.8 (108.7) | 2.8 (20.1) | 1071.0 (4255.4) |
| Drinking water availability**^§^** | | | |
| Never/sometimes | 56.6 (391.4) | 11.6 (91.1) | 1572.4 (5390.9) |
| Always | 75.7 (272.7) | 13.8 (116.1) | 1243.2 (4272.2) |
| Tissue/water for anal cleansing availability**^§^** | | | |
| Never/sometimes | 63.3 (376.5) | 12.5 (100.3) | 1598.6 (5313.9) |
| Always | 27.9 (190.9) | 5.7 (29.4) | 0.9 (12.8) |
| Latrine sanitation: cleanliness^*^ | | | |
| 1^st^ quartile | 57.6 (454.7) | 20.9 (99.7) | 2258.6 (6370.3) |
| 2^nd^ quartile | 77.3 (254.8) | 10.7 (105.5) | 1384.3 (4753.6) |
| 3^rd^ quartile | 22.0 (124.0) | 3.5 (32.9) | 1351.2 (5077.9) |
| 4^th^ quartile | 89.2 (506.1) | 13.5 (126.8) | 967.9 (4001.9) |
| Latrine sanitation: structural integrity^*^ | | | |
| 1^st^ quartile | 67.1 (459.7) | 3.1 (22.6) | 1589.0 (5495.3) |
| 2^nd^ quartile | 28.4 (133.3) | 11.5 (67.8) | 1765.0 (5858.6) |
| 3^rd^ quartile | 63.7 (243.6) | 23.5 (173.1) | 1044.7 (4005.3) |
| 4^th^ quartile | 86.0 (494.2) | 11.9 (71.8) | 1533.3 (4914.0) |

^†^Improved sources are defined by the UNICEF/WHO joint monitoring programme (wssinfo.org). ^§^school-aggregated proportion of pupil-reported availability. ^*^A higher quartile indicates better cleanliness/structural integrity.
